# Phenprocoumon Dose Requirements, Dose Stability and Time in Therapeutic Range in Elderly Patients With *CYP2C9* and *VKORC1* Polymorphisms

**DOI:** 10.3389/fphar.2019.01620

**Published:** 2020-01-28

**Authors:** Katharina Luise Schneider, Melanie Kunst, Ann-Kristin Leuchs, Miriam Böhme, Klaus Weckbecker, Kathrin Kastenmüller, Markus Bleckwenn, Stefan Holdenrieder, Christoph Coch, Gunther Hartmann, Julia Carolin Stingl

**Affiliations:** ^1^ Research Division, Federal Institute for Drugs and Medical Devices, Bonn, Germany; ^2^ Centre for Translational Medicine, Medical Faculty of the University of Bonn, Bonn, Germany; ^3^ Institute of General Practice, Medical Faculty of the University of Düsseldorf, Düsseldorf, Germany; ^4^ Institute of General Practice and Family Medicine, Medical Faculty of the University of Bonn, Bonn, Germany; ^5^ Institute of Laboratory Medicine, German Heart Centre Munich, Munich, Germany; ^6^ Institute of Clinical Chemistry and Clinical Pharmacology, Medical Faculty of the University of Bonn, Bonn, Germany

**Keywords:** CYP2C9, dose, international normalized ratio, phenprocoumon, VKORC1, time in therapeutic range

## Abstract

**Background:**

Dose requirements of vitamin K antagonists are associated with *CYP2C9* and *VKORC1*, but, compared to warfarin, less data is available about phenprocoumon. Furthermore, the effects on dose stability and anticoagulation quality are still unclear.

**Methods:**

Aim was to scrutinize phenprocoumon dose requirements, dose stability and anticoagulation quality in association to *CYP2C9* and *VKORC1* in a natural cohort of elderly primary care patients. As a subgroup within the IDrug study, phenprocoumon treated patients with at least two INR values within three months before enrollment (n = 209) were analyzed concerning average weekly dose, standard deviation of weekly dose (intra-subject variability), constant dose (yes/no), average INR and TTR grouped by *CYP2C9* and *VKORC1* (and combinations).

**Results:**

Average weekly dose per patient was 14.4 ± 5.3 mg, 11.9 ± 4.0 mg and 11.2 ± 4.3 mg in *CYP2C9* wildtypes, *2 and *3 carriers (p < .0001) and 16.0 ± 4.2 mg, 13.3 ± 5.1 mg and 8.0 ± 2.7 mg per week in *VKORC1* CC, CT and TT genotypes, respectively (p < .0001). Significant differences concerning intra-subject variability were detected among all groups (p < .0001) with the smallest variability in CYP2C9*3 carriers. TTR medians were 75.4%, 79.4% and 100% in wildtypes, *2 and *3 carriers, respectively (p = 0.0464). The proportion of patients with perfect control was highest among *3 carriers, but this result was not significant (p = 0.0713).

**Discussion:**

Our analyses support the results of previous investigations regarding genotype-associated dose requirements and raise the hypothesis that dose stability and anticoagulation quality may be increased in CYP2C9*3 carriers. However, our data should be treated cautiously due to the small sample size.

**Clinical Trial Registration:**

German Clinical Trials Register, identifier DRKS00006256.

## Introduction

Vitamin K antagonists are widely used drugs for anticoagulation in medical conditions such as atrial fibrillation and venous thromboembolism. Anticoagulation quality can be measured by regular international normalized ratio (INR) checks (Ansell et al., 2008). While undercoagulation is associated with an elevated risk of (recurrent) ischemic events, overanticoagulation can substantially increase the risk of bleeding (Merli and Tzanis, 2009). To avoid this, the recommended therapeutic range by the American Heart Association is an INR of 2–3 for the most frequent indications (Proceedings of the American College of Chest Physicians 5th Consensus on Antithrombotic Therapy, 1998).

Among others, pharmacogenetic factors have been described to be associated with dose requirements in vitamin K antagonists. Two genes of interest in this context are *CYP2C9* with the alleles *2 and *3 and *VKORC1 1173C > T.* CYP2C9 is involved in the metabolism of vitamin K antagonists. *2 and *3 variant carrier status has been associated with a lower metabolic capacity (Kirchheiner et al., 2004; Ufer et al., 2004). VKORC1 is the drug target of vitamin K antagonists and carriers of the *1173C > T* variant have been described to have a higher sensitivity for these drugs (Reitsma et al., 2005).

Data availability concerning clinical utility of genotyping varies among the different coumarin derivatives. In contrast to warfarin, which has been widely studied and for which a genotype-guided dosing algorithm has been shown to be beneficial in the randomized controlled EU-PACT trial (Pirmohamed et al., 2013), there is considerably less data available concerning phenprocoumon. The EU-PACT trials on phenprocoumon and acenocoumarol had to be combined for analysis due to recruitment issues and did not show a significant effect of genotype-guided dosing for these two substances (Verhoef et al., 2013). In two more recent publications the authors performed sub-analyses within the phenprocoumon cohort. They observed that the algorithm may have predicted the dose overcautiously in the VKORC1 AA–CYP2C9*1/*1 subgroup (Baranova et al., 2017) and that the algorithm in general should be revised for older patients ≥75 years (Zhang et al., 2017). The Royal Dutch Pharmacists Association—Pharmacogenetics Working Group recommends a reduced initial dose and more frequent INR checks in homozygous *VKORC1* mutation carriers, but no specific action for *CYP2C9* variant carriers, although these variants could result in a reduction of the maintenance dose, as well (Dutch Pharmacogenetics Working Group Guidelines August 2019, 2019). To date, it is unclear in what way these pharmacogenetic variants affect dose stability and anticoagulation quality. Objective of the present analysis was to investigate phenprocoumon dose requirements, dose variability and anticoagulation quality in association to *CYP2C9* and *VKORC1* in a natural cohort of elderly primary care patients.

## Methods

### Patient Population

The analyzed patient population was the subgroup of patients treated with phenprocoumon taken from the IDrug study cohort, a two-arm, multicenter, randomized, controlled trial on individualized risk assessment in high risk patients for adverse drug reactions (Stingl et al., 2016). Patients were recruited by their general practitioners between October 2014 and March 2017. Inclusion criteria for the IDrug study were: age ≥60 years, multimorbidity with at least two diagnoses, and long-term drug treatment with one (or more) antithrombotic drug(s) and with at least one additional drug. A detailed description of the trial design has been published previously (Stingl et al., 2016). Either the general practitioners or the patients themselves documented the INR values and daily/weekly doses in a paper-based diary (usually immediately after INR measurement). In other cases these data were part of the electronic patient record. Upon enrollment, patients on vitamin K antagonists and their general practitioners were asked to provide dosing and INR information of the previous three months because this was the required minimum length of anticoagulation treatment before study inclusion. This documentation of INR and phenprocoumon dosing prior to the inclusion in the IDrug study was the basis of our analyses. All patients from whom at least two INR values were available were included.

### Genotyping

As a part of the IDrug study procedures, a blood sample was taken from all patients upon enrollment. DNA extraction and genotyping were performed by the Institute of Clinical Chemistry and Clinical Pharmacology, University Bonn. Genotyping included rs1799853 (CYP2C9*2), rs1057910 (CYP2C9*3) and rs9934438 (*VKORC1 1173C > T*). Melting curve analyses of fluorescent Real-Time-PCR amplification products were used (“Light Mix Kits” used as advised by the manufacturer) for allelic identification. If no mutation was detected, wildtype carrier status was assumed. We used a goodness-of-fit χ^2^ test to evaluate agreement with Hardy–Weinberg expectations.

### Statistical Analysis

The analysis was based on all INR values collected in the week a patient was enrolled and within the 12 weeks prior to the week of enrollment (i.e. up to 13 weeks per patient), and hence, the relevant observation period per patient ranges from the week with the first to the week with the last available INR measurement. Information on patients’ weekly doses beginning one week before the first INR value were included in the analysis to account for the fact that INR values are influenced by the previously applied dosage. With regard to *VKORC1,* comparisons were based on genotype (CC, CT and TT). For *CYP2C9,* two different grouping approaches were pursued: a) grouping by *CYP2C9* allele carrier status, i.e. wildtypes (*1/*1), *2 carriers (*1/*2, *2/*2) and *3 carriers (*1/*3, *2/*3, *3/*3) (Schalekamp et al., 2007; van Schie et al., 2012) and b) grouping by *CYP2C9* phenotype, i.e. extensive (*1/*1), intermediate (*1/*2, *1/*3) and poor metabolizers (*2/*2, *2/*3, *3/*3). *CYP2C9* phenotypes were all genetically inferred and not experimentally measured. Additionally, three different combinations were considered to investigate gene–gene-interactions: a) *VKORC1* genotype + *CYP2C9* genotype, b) *VKORC1* genotype + *CYP2C9* allele carrier status, c) *VKORC1* genotype + *CYP2C9* phenotype.

The Jonckheere–Terpstra test (one-sided) was used to determine whether there is a declining trend in the average weekly dose between the aforementioned *VKORC1* and *CYP2C9* groups and group combinations. Their presumptive order for this declining trend regarding *VKORC1* genotypes and *CYP2C9* geno-/phenotypes was predefined based on the results by van Schie et al. expecting a more pronounced effect of *VKORC1* than of *CYP2C9* (van Schie et al., 2011). The genetic groups and group combinations are sorted by this expected order in **Table 1** and the **Tables S3–S6**. All following tests were two-sided. Two different approaches were pursued to investigate dose stability. To analyze the standard deviation of weekly doses per patient (i.e. the within-subject variability), a mixed model for repeated measurements including a fixed effect for the *CYP2C9/VKORC1* grouping variable, a random subject effect (between-subject variability) and unequal residual variances (within-subject variability) per *CYP2C9/VKORC1* group was applied. Based on this model (and a simplified one assuming equal residual variances across *CYP2C9/VKORC1* groups), a likelihood ratio test was used to test the null hypothesis of equal within-subject variability between *CYP2C9/VKORC1* groups. Additionally, we investigated the association between the genetic groups and a binary variable ‘constant dose’ indicating whether the patient remained on a stable weekly dose (i.e. no dose changes) during the observation period (‘constant dose’ = yes) or changed his weekly dose at least once (‘constant dose’ = no) with Fisher’s exact test. To compare the average INR across groups and group combinations an analysis of variance (ANOVA) was performed. Time in therapeutic range (TTR) was calculated using the Rosendaal method (linear interpolation between two INR measurements) (Rosendaal et al., 1993) and compared between the genetic groups using Kruskal–Wallis test. Furthermore, TTR was compared between patients on constant and patients on variable weekly doses with a Wilcoxon–Mann–Whitney test. We also pursued a categorical approach based on the methods by Farsad et al. (Farsad et al., 2016) to analyze TTR, which we modified by adding the categories “perfect control” and “no control”. Our TTR categories were defined as follows: perfect control: TTR = 100%, good control: 70% ≤ TTR < 100%, moderate control: 50% < TTR < 70%, poor control: 0% < TTR ≤ 50%, no control: TTR = 0%. The categorized TTRs were compared between the groups with Fisher’s exact test.

**Table 1:** Genotype distribution, dose requirements, INR, dose stability and TTR.

Genetic Group^a^	No. of Patients	Average Weekly Dose per Patient (mg)^b^ mean ± SD	Average INR mean ± SD	Standard Deviation of Weekly Dose per Patient (mg)^c^ mean ± SD	Constant Dose “yes”^c^ *n* (%)	TTR (%) median
All patients	209	13.6 ± 5.1	2.4 ± 0.4	1.18 ± 1.51	47 (22.5)	77.2
*CYP2C9* allele carrier status						
Wildtype	145	14.4 ± 5.3	2.3 ± 0.3	1.27 ± 1.48	30 (20.7)	75.4
*2 carrier	37	11.9 ± 4.0	2.4 ± 0.4	1.29 ± 1.95	6 (16.2)	79.4
*3 carrier	27	11.2 ± 4.3	2.4 ± 0.3	0.52 ± 0.53	11 (40.7)	100.0
p-value^d^		<.0001	0.4038	<.0001	0.0737	0.0464
*VKORC1* genotype						
CC	76	16.0 ± 4.2	2.4 ± 0.4	1.27 ± 1.53	15 (19.7)	77.5
CT	106	13.3 ± 5.1	2.3 ± 0.3	1.13 ± 1.58	27 (25.5)	77.1
TT	27	8.0 ± 2.7	2.4 ± 0.3	1.07 ± 1.16	5 (18.5)	77.1
p-value^d^		<.0001	0.4841	<.0001	0.5278	0.8844

SD, standard deviation; INR, International Normalized Ratio; TTR, time in therapeutic range. Based on all available data. ^a^ Genetic groups are sorted by the expected weekly dose in descending order. ^b^ Four patients were excluded due to missing dose information. ^c^ Four patients with missing dose information and two patients with only one dose available were excluded. ^d^ p-value based on Jonckheere–Terpstra trend test (average weekly dose per patient), ANOVA (average INR), likelihood ratio test (standard deviation of weekly dose per patient), Fisher’s exact test (constant dose) or Kruskal–Wallis test (TTR).

Sensitivity analyses were conducted to evaluate the influence of missing dose information. In particular, the presented analyses were also conducted based on only those subjects with complete dose information. We did not know the exact start of anticoagulation treatment but the vast majority of the patients could be attributed to the maintenance phase as they provided more retrospective data than the required three months (the rest could not be clearly attributed). In order to check for potential bias due to unequally distributed patients with more recent anticoagulation initiation, we compared the length of documentation of all available data before enrollment between genetic groups. Statistical analyses were conducted with SAS 9.3 (SAS Institute Inc). P-values ≤ 0.05 were considered statistically significant for all two-sided tests and ≤0.025 for the one-sided trend tests. However, no adjustment for multiplicity was done and all results are exploratory.

## Results

### Description of the Patient Cohort

The analyzed patient population consisted of 209 individuals. Mean age was 75.6 years (SD: 6.5). One hundred and twenty-two (58.4%) were men and 87 (41.6%) were women. The length of observation period (number of weeks from week with first to week with last INR measurement) was four to six weeks in 10 (4.8%), seven to nine weeks in 43 (20.6%), 10–12 weeks in 115 (55.0%) and 13 weeks in 40 (19.1%) patients. In only one case it comprised less than four weeks. The mean length of the observation period was 10.6 2.1 weeks (SD: 2.1). For most patients three to four (45.5%) or five to six (22.0%) INR measurements were collected during the observation period. The number of INRs per week was on average 0.53 (SD: 0.40). In 181 (86.6%) patients all dosage information was available. In four patients (1.9%) all dosage information was missing. These four patients were excluded from all analyses concerning dosage. Two patients with only one weekly dose available were excluded from analyses regarding dose variability. The number of INR measurements, length of the observation period and dose availability for the different genetic groups can be found in the supplement (**Table S1**). Data availability was comparable among all groups. Median length of documentation was 303 days, when evaluating all available data, and did not differ substantially between the genetic groups.

### Genotyping Results

Seventy-six (36.4%) of the patients were *VKORC1* wildtypes (CC), 106 (50.7%) were heterozygous (CT) and 27 (12.9%) were homozygous (TT) mutation carriers. With regard to *CYP2C9* 145 (69.4%) patients had the genotype *1/*1, meaning their allele carrier status was “wildtype” and their phenotype was “extensive”. Grouping for allele carrier status revealed 37 *2 carriers (17.7%) and 27 *3 carriers (12.9%). In the “intermediate” and the “poor” phenotype group were 59 (28.2%) and five patients (2.4%), respectively. All these genotyping results were in Hardy–Weinberg equilibrium. *VKORC1* genotypes and *CYP2C9* allele carrier status are displayed in **Table 1**. The distribution of the *CYP2C9* phenotypes and all group combinations can be found in the supplement (**Tables S3–S6**). Genotyping results were similarly distributed in the subgroup of patients with complete dose information as compared to the overall population.

### Average Weekly Dose and Average INR

Descriptive statistics for the average weekly dose per patient are depicted in **Table 1**. The mean ± SD of the average weekly dose per patient was 14.4 ± 5.3 mg in the *CYP2C9* wildtype group, and 11.9 ± 4.0 mg and 11.2 ± 4.3 mg in *2 carriers and *3 carriers, respectively. Differences were more distinct between the *VKORC1* groups: 16.0 ± 4.2 mg, 13.3 ± 5.1 mg and 8.0 ± 2.7 mg for CC, CT and TT genotypes, respectively. For *CYP2C9* phenotypes and group combinations results are displayed in the **Supplementary Material**. All genetic groups and group combinations showed a significantly declining trend concerning the average weekly dose per patient (all p < .0001).

No significant differences among the genetic groups regarding average INR per patient were detected. The means of the average INRs were between 2.3 and 2.5 with standard deviations of 0.3–0.4, so the values were very similar among all groups. Average weekly doses and INRs within the subgroup of patients with complete dose information were comparable to those in the whole cohort.

### Dose Stability

Looking at the standard deviation of the weekly dose per patient across the allele carrier status groups, a particularly small standard deviation (0.52 ± 0.53 mg) and a high proportion of patients on constant doses (40.7%) were seen in *3 carriers compared to wildtypes and *2 carriers. The standard deviation of the weekly dose per patients was significantly different between *CYP2C9* groups (p < .0001), also when only patients with complete dose information were included. The differences regarding constant doses (yes/no) did not reach statistical significance (p = 0.0737). Among *VKORC1* genotypes, a declining trend of within-subject dosing variability was also observed and the difference between groups was significant (p < .0001) in the whole cohort as well as in patients with complete dose information. However, the effect was less pronounced than among *CYP2C9* groups and no significant differences concerning the proportion of patients with constant dose were seen (p = 0.5278). Detailed results concerning dose stability can be found in **Table 1**. Grouping by *CYP2C9* phenotype produced similar results as grouping by allele carrier status (**Table S3**).

### Time in Therapeutic Range

**Figure 1:**
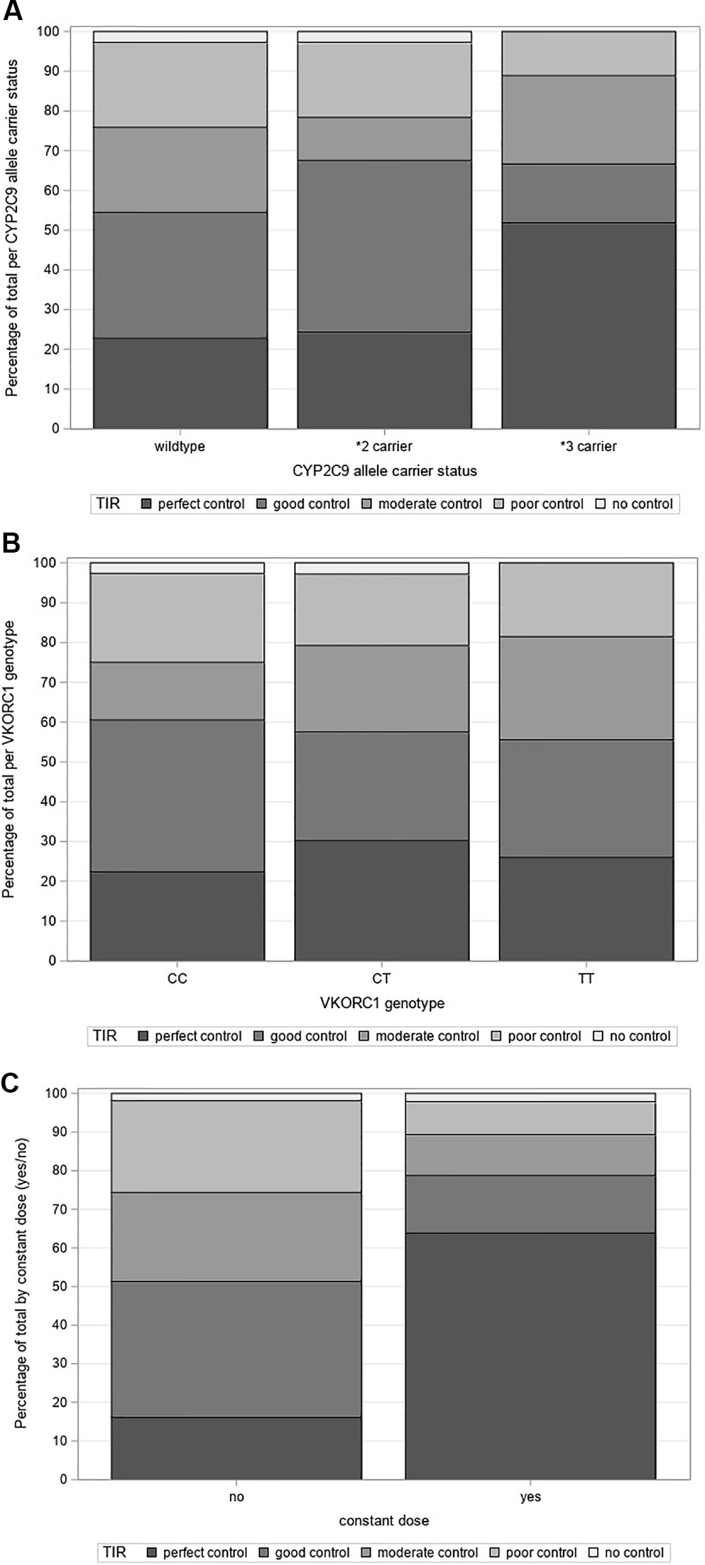
**(A)** Time in therapeutic range grouped by *CYP2C9* allele carrier status, **(B)** Time in therapeutic range grouped by *VKORC1* genotype, **(C)** Time in therapeutic range grouped by “constant dose (yes/no)”.

Grouping by *CYP2C9* allele carrier status revealed a significant difference regarding TTR among the groups (p = 0.0464). Median TTRs were 75.4%, 79.4% and 100.0% for wildtypes, *2 and *3 carriers, respectively. Sensitivity analysis based on patients with complete dose information showed very similar median TTRs. The categorical analysis showed that the proportion of patients with perfect control was higher among *3 carriers (51.9%) than among *2 carriers (24.3%) and wildtypes (22.8%), which is illustrated in **Figure 1A**). However, the results of the categorical comparison did not reach significance (p = 0.0713). There were no significant TTR differences between *VKORC1* genotypes (p = 0. 7060) (**Figure 1B**). Patients on a constant dose had significantly better TTRs than patients on a variable dose with medians of 100.0% vs. 71.3% (p < .0001) and a higher proportion of patients with perfect control with 63.8% vs. 16.0% (**Figure 1C**) (p < .0001). Poor and intermediate *CYP2C9* phenotypes spent in median more time within therapeutic range than extensive metabolizers (see **Table S3**), but these differences were not significant (p = 0.1294).

## Discussion

In the present analysis, we evaluated the influence of *CYP2C9* and *VKORC1* pharmacogenetic factors on INR adapted dosing, dose stability and anticoagulation quality of phenprocoumon in a group of elderly phenprocoumon treated outpatients in a natural setting. We detected differences concerning average weekly dose between individuals in the *CYP2C9* and *VKORC1* genotype groups and associations between *CYP2C9* allele carrier status and intra-individual dose stability as well as anticoagulation quality (time spent in therapeutic range). Genotype based differences concerning average weekly doses were detected for both, *CYP2C9* and *VKORC1* alleles, and were more pronounced between *VKORC1* genotype groups than across *CYP2C9* groups. Our genotyping results (Verhoef et al., 2013; Schalekamp et al., 2007) as well as our observed weekly doses (Puehringer et al., 2010; Qazim et al., 2009; Teichert et al., 2011) are in line with other publications. To ensure that the described dose differences between the genotype groups were not a result of differences in general INR levels (e.g. to check that *VKORC1* TT patients achieved generally similar INR values as *VKORC1* CC patients but with lower doses), we looked at the average INR per patient and mean and standard deviation were similar across groups (**Table 1**). Means of average INRs were 2.3–2.5 with standard deviations of 0.3–0.4 in all genetic groups, which is in concordance with the recommended INR range of 2–3 (Kirchhof et al., 2017). Data on dose requirements combining *CYP2C9* and *VKORC1* are relatively scarce, but their reported average weekly doses are overall comparable to our results (Puehringer et al., 2010; Abduljalil et al., 2013). In our analysis, a significant declining trend in the average weekly dose per patient was observed across the predefined order of group combinations, even though some of the groups contained only a small number of patients.

Comparing the standard deviations of the weekly dose per patient (intra-individual variability), we observed the smallest variability in CYP2C9*3 carriers. The standard deviation of weekly dose per patient was 0.52 mg per week for *3 carriers compared to 1.27 mg for wildtypes. Additionally, *3 carriers were observed to receive constant phenprocoumon doses more frequently than wildtypes or *2 carriers, but this results did not reach statistical significance (p = 0.0737).

Time in therapeutic range was significantly different among the *CYP2C9* allele carrier groups with better TTRs in *2 and particularly in *3 carriers than in wildtypes (p = 0.0464). The categorical approach did not reach significance, but also demonstrated a better anticoagulation quality of the *3 carriers with a high proportion of patients with perfect control. We also found a significant positive association between constant doses and TTR (p < .0001). At first sight, this association appears to be trivial, as doctors would probably stick to the previous dose if the INR value lies within the therapeutic range. However, clinical experiences show that maintenance of an unchanged dose does not necessarily lead to constant INRs (in that case regular INR checks would be obsolete). Instead, clinical and lifestyle factors (e.g. food or concomitant drug intake) can interact with oral anticoagulants and their efficacy. With our analysis of intra-individual dose variability and TTR, we sought to investigate whether patients with genetic polymorphisms differ from wildtypes concerning dose stability and anticoagulation quality. The association between constant doses and TTR may be attributable to the fact that *3 carriers performed well in both categories.

To sum up, in our cohort of 209 patients on phenprocoumon therapy, *CYP2C9* and *VKORC1* polymorphisms were accompanied by lower average weekly doses. Additionally, *3 carriers of *CYP2C9* seemed to have more stable doses and spend more time within therapeutic range in our exploratory analysis. During routine treatment with phenprocoumon in outpatient conditions, dosing was achieved with better stability in CYP2C9*3 carriers than in wildtypes. There are only few studies investigating the association of pharmacogenetic factors and phenprocoumon anticoagulation quality in patients without previous intervention (e.g. dosing according to a genotype-guided algorithm). We found two studies indicating a negative effect of *CYP2C9* polymorphisms (Schalekamp et al., 2007; Nagler et al., 2017) and one study that did not detect a significant association (Brehm et al., 2016). In contrast, in an analysis by Verhoef et al. CYP2C9*2 and *3 carriers spent significantly less time below therapeutic range. However, in this publication *3 carriers also showed the longest time above range and the data include patients with a genotype-guided intervention. Additionally, the differences in out-of-range INRs were only detected within the first month of treatment in the study by Verhoef et al. (Verhoef et al., 2012), whilst the vast majority of our patients was already in their maintenance phase (the rest could not be clearly attributed). Another study indicating a possible positive effect of *CYP2C9* polymorphisms has been published by Luxembourg et al. They reported that CYP2C9*2 and *3 carriers reached stable INRs faster than wildtypes with fewer anticoagulation clinic visits in the initiation phase (Luxembourg et al., 2011). At first sight, these results appear to be counterintuitive, as this would indicate *CYP2C9* polymorphisms to be an advantage rather than a disadvantage in phenprocoumon patients. However, until now this association is not fully understood and there may be arguments underlining this hypothesis. Phenprocoumon has a longer half-life compared to warfarin and acenocoumarol (Ufer, 2005) and has been shown to produce a more stable anticoagulation (Fihn et al., 2003). Consequently, it may be hypothesized that an additionally slower metabolism due to *CYP2C9* polymorphisms could enhance this effect, also because the lower dosages in polymorphism carriers are expected to result in smaller peak/trough fluctuations. This could possibly explain both, stable dose requirements and better TTRs observed in this analysis.

There are different possibilities to group *CYP2C9* genotypes and to date it is unclear which way achieves better discrimination. When comparing our results grouped by allele carrier status and by phenotype, the results concerning average weekly dose were quite similar, so both approaches seem to discriminate equally well in this category. When evaluating the TTR, the results were more distinct between the allele carrier status groups than the phenotypes. In general, grouping by *CYP2C9* phenotypes has the disadvantage of a fairly small poor metabolizer group. This might be the reason why studies grouping for phenotypes can hardly be found in the literature. All in all, our results regarding phenotype showed the same trends as those regarding allele carrier status.

When interpreting our data, a few limitations should be considered. The analyzed population was a subgroup of the IDrug study and, even though phenprocoumon was the most frequently prescribed anticoagulant, the number of patients was relatively small. Therefore, our results should be treated cautiously because of the small group sizes in the rare allelic genotype groups. Our study was conducted in Germany and patients were at least 60 years old, so generalizability to other ages and ethnicities may be limited. However, phenprocoumon is predominantly used in older patients in Europe. Due to the pragmatic design of the IDrug study, data were collected in a setting of everyday practice. The analysis is based on patient self-reported diaries or data entries by the general practitioners as there is no consistently used method of documentation in daily routine. This led to variabilities in data quality including missing doses and INR values. However, we illustrated data availability in **Table S1** and did not detect any substantial differences. Notably, there were only few patients with only two INR values available within the smallest groups, i.e. *CYP2C9* poor metabolizers, CYP2C9*3 carriers and *VKORC1* TT genotypes (see **Tables S1** and **S2** in the supplement). We also performed sensitivity analyses only using data of patients with complete dose information, which was of particular interest with regard to dose stability analyses. The results were comparable to those based on data of all patients. While we did not know the exact beginning of anticoagulation therapy of each patient, we knew that the majority was not in the initiation phase. Furthermore, differences regarding more recent initiation of anticoagulation treatment between the genetic groups were not apparent, so we could exclude potential bias due to unequally distributed patients with more recent anticoagulation initiation. When interpreting our data, it needs to be considered that we deliberately refrained from adjusting for any covariates (e.g. interacting drugs) to evaluate the overall rather than the independent effect of the genotypes. We considered this as the more clinically relevant effect. It may be of interest for further research how this overall effect relates to direct and indirect effects in our naturalistic cohort. However, given the limited sample size this analysis may not provide very robust/reliable conclusions. A common limitation of pharmacogenetic studies is that genotyping often only includes the most frequent currently known alleles, so carriers of rare alleles may falsely be labeled as wildtypes. This could be the case in our analysis, as well. However, a meta-analysis of population scale sequencing projects suggests that variants other than *2 and *3 are rare in Europeans (Zhou et al., 2017). Additionally, we only used data up until enrollment for this analysis, as the IDrug study was still running when this analysis was conducted. Genotyping results were unknown to patients and general practitioners at enrollment so that data analyzed where not influenced by genotype knowledge. After enrollment, test group patients and their treating physicians received a patient tailored drug risk assessment leaflet including pharmacogenetic information whilst control group patients only received a standardized leaflet (Stingl et al., 2016). After trial close out it is planned to subsequently investigate whether knowledge of the patient’s genotype had an influence on dosing and TTR as well as adverse effects such as bleeding or thromboembolic events.

## Data Availability Statement

All datasets generated and analyzed for this study are included in the article/**Supplementary Material**.

## Ethics Statement

All procedures performed in studies involving human participants were in accordance with the ethical standards of the Ethics Committees of the University of Bonn, of the Medical Association of North Rhine and of the Medical Association of Rhineland-Palatinate and with the 1964 Helsinki declaration and its later amendments or comparable ethical standards.

## Author Contributions

KS was the coordinator of the IDrug study, involved in acquisition, analysis and interpretation of the data and wrote the manuscript. MK was involved in data acquisition, analysis and interpretation. MB performed data management and analysis. AL was responsible for statistical analyses and data interpretation. KK, MB and KW were involved in recruitment and data acquisition. SH, CC and GH performed genotyping. JS conceived the IDrug study and was involved in acquisition, analysis and interpretation of the data. All authors read and approved the manuscript.

## Funding

The IDrug study is financially supported by a research grant from the Federal Ministry of Education and Research (grant number 01GY1333A).

## Conflict of Interest

The authors declare that the research was conducted in the absence of any commercial or financial relationships that could be construed as a potential conflict of interest.
