# Supplementary material for: Phenprocoumon Dose Requirements, Dose Stability and Time in Therapeutic Range in Elderly Patients With CYP2C9 and VKORC1 Polymorphisms
Source: Front Pharmacol. 2020 Jan 28;10:1620. doi: 10.3389/fphar.2019.01620 (PMC6997201; doi:10.3389/fphar.2019.01620)
Supplement: Supplementary file 1 [file DataSheet_1.docx]

**Table S1** Data availability among genotype groups

|  | No. of patients, *n* (%) | *CYP2C9* allele carrier status, *n* (%) | | | *VKORC1* genotype, *n* (%) | | |
| --- | --- | --- | --- | --- | --- | --- | --- |
|  |  | Wildtype | **2* carrier | **3* carrier | CC | CT | TT |
| No. of INR measurements | | | | | | | |
| 2 | 15 (7.2) | 9 (6.2) | 4 (10.8) | 2 (7.4) | 6 (7.9) | 7 (6.6) | 2 (7.4) |
| 3-4 | 95 (45.5) | 64 (44.1) | 15 (40.5) | 16 (59.3) | 33 (43.4) | 50 (47.2) | 12 (44.4) |
| 5-6 | 46 (22.0) | 30 (20.7) | 11 (29.7) | 5 (18.5) | 20 (26.3) | 23 (21.7) | 3 (11.1) |
| 7-8 | 26 (12.4) | 20 (13.8) | 4 (10.8) | 2 (7.4) | 9 (11.8) | 12 (11.3) | 5 (18.5) |
| ≥9 | 27 (12.9) | 22 (12.9) | 3 (8.1) | 2 (7.4) | 8 (10.5) | 14 (13.2) | 5 (18.5) |
| No. of weeks from week with first to week with last INR (observation period) | | | | | | | |
| <4 | 1 (0.5) | 0 (0.0) | 0 (0.0) | 1 (3.7) | 0 (0.0) | 1 (0.9) | 0 (0.0) |
| 4-6 | 10 (4.8) | 6 (4.1) | 3 (8.1) | 1 (3.7) | 3 (3.9) | 6 (5.7) | 1 (3.7) |
| 7-9 | 43 (20.6) | 27 (18.6) | 7 (18.9) | 9 (33.3) | 15 (19.7) | 20 (18.9) | 8 (29.6) |
| 10-12 | 115 (55.0) | 81 (55.9) | 21 (56.8) | 13 (48.1) | 37 (48.7) | 64 (60.4) | 14 (51.9) |
| 13 | 40 (19.1) | 31 (21.4) | 6 (16.2) | 3 (22.2) | 21 (27.6) | 15 (14.2) | 4 (14.8) |
| Dose availability (proportion of weeks with available dose information) | | | | | | |  |
| 100% | 181 (86.6) | 126 (86.9) | 31 (83.8) | 24 (88.9) | 68 (89.5) | 91 (85.5) | 22 (81.5) |
| ≥50% - <100% | 17 (8.1) | 9 (6.2) | 5 (13.5) | 3 (11.1) | 5 (6.6) | 9 (8.5) | 3 (11.1) |
| >0% - <50% | 7 (3.3) | 7 (4.8) | 0 (0.0) | 0 (0.0) | 2 (2.6) | 3 (2.8) | 2 (7.4) |
| 0% | 4 (1.9) | 3 (2.1) | 1 (2.7) | 0 (0.0) | 1 (1.3) | 3 (2.8) | 0 (0.0) |
| ∑ | 209 (100) | 145 (100) | 37 (100) | 27 (100) | 76 (100) | 106 (100) | 27 (100) |

**Table S2** Data availability among CYP2C9 phenotype groups

|  | All patients, *n* (%) | CYP2C9 phenotype, *n* (%) | | | | |
| --- | --- | --- | --- | --- | --- | --- |
|  |  | Normal | Intermediate | | | Poor |
| No. of INR measurements | | | | | | |
| 2 | 15 (7.2) | 9 (6.2) | | 6 (10.2) | 0 (0) | |
| 3-4 | 95 (45.5) | 64 (44.1) | | 29 (49.2) | 2 (40.0) | |
| 5-6 | 46 (22.0) | 30 (20.7) | | 15 (25.4) | 1 (20.0) | |
| 7-8 | 26 (12.4) | 20 (13.8) | | 5 (8.5) | 1 (20.0) | |
| ≥9 | 27 (12.9) | 22 (15.2) | | 4 (6.8) | 1 (20.0) | |
| No. of weeks from week with first to week with last INR (observation period) | | | | | | |
| <4 | 1 (0.5) | 0 (0.0) | | 1 (1.7) | 0 (0.0) | |
| 4-6 | 10 (4.8) | 6 (4.1) | | 4 (6.8) | 0 (0.0) | |
| 7-9 | 43 (20.6) | 27 (18.6) | | 15 (25.4) | 1 (20.0) | |
| 10-12 | 115 (55.0) | 81 (55.9) | | 32 (54.2) | 2 (40.0) | |
| 13 | 40 (19.1) | 31 (21.4) | | 7 (11.9) | 2 (40.1) | |
| Dose availability (proportion of weeks with available dose information) | | | | | | |
| 100% | 181 (86.6) | 126 (86.9) | | 52 (88.1) | 3 (60.0) | |
| ≥50% - <100% | 17 (8.1) | 19 (13.1) | | 6 (10.2) | 2 (40.0) | |
| >0% - <50% | 7 (3.3) | 7 (4.8) | | 0 (0.0) | 0 (0.0) | |
| 0% | 4 (1.9) | 3 (2.1) | | 1 (1.7) | 0 (0.0) | |
| ∑ | 209 (100) | 145 (100) | | 59 (100) | 5 (100) | |

INR, International Normalized Ratio

**Table S3** Genotype distribution, dose requirements, INR, dose stability and TTR among CYP2C9 phenotype groups

| Genetic group^a^ | No. of patients | Average weekly dose per patient (mg) ^b^  mean ± SD | Average INR  mean ± SD | Standard deviation of weekly dose per patient (mg) ^c^  mean ± SD | Constant dose “yes” ^c^ *n* (%) | TTR (%)  median |
| --- | --- | --- | --- | --- | --- | --- |
| All patients | 209 | 13.6±5.1 | 2.4±0.4 | 1.18±1.51 | 47 (22.5) | 77.2 |
| CYP2C9 phenotype | | | | | | |
| Normal | 145 | 14.4±5.3 | 2.3±0.3 | 1.27±1.48 | 30 (20.7) | 75.4 |
| Intermediate | 59 | 11.7±4.1 | 2.4±0.4 | 1.01±1.61 | 14 (23.7) | 81.0 |
| Poor | 5 | 11.0±4.2 | 2.5±0.3 | 0.38±0.58 | 3 (60.0) | 84.7 |
| p-value^d^ |  | <.0001 | 0.3927 | <.0001 | 0.1364 | 0.1294 |

SD, standard deviation; INR, International Normalized Ratio; TTR, time in therapeutic range.

Table based on all available data. ^a^ Genetic groups are sorted by the expected weekly dose in descending order. ^b^ Four patients were excluded due to missing dose information. ^c^ Four patients with missing dose information and two patients with only one dose available were excluded. ^d^ p-value based on Jonckheere-Terpstra trend test (average weekly dose), ANOVA (average INR), likelihood ratio test (standard deviation of weekly dose per patient), Fisher’s exact test (constant dose) or Kruskal-Wallis test (TTR)

**Table S4** Average weekly dose per patient grouped by a combination of CYP2C9 phenotype + *VKORC1* genotype

| CYP2C9 phenotype | *VKORC1* genotype | No. of patients | Average weekly dose per patient (mg)  mean ± SD |
| --- | --- | --- | --- |
| Normal | CC | 54 | 16.5±4.0 |
| Intermediate | CC | 20 | 14.5±4.6 |
| Poor | CC | 2 | 14.5±1.5 |
| Normal | CT | 74 | 14.5±5.3 |
| Intermediate | CT | 30 | 10.5±3.2 |
| Poor | CT | 2 | 10.6±0.2 |
| Normal | TT | 17 | 7.3±2.5 |
| Intermediate | TT | 9 | 9.5±2.6 |
| Poor | TT | 1 | 4.5 |
| p-value^b^ |  |  | <.0001 |

Based on all available data. ^a^ Four patients without dose information available were excluded. ^b^ p-value based on Jonckheere-Terpstra trend test

**Figure S1** Average weekly dose per patient grouped by a combination of CYP2C9 phenotype + *VKORC1* genotype

**
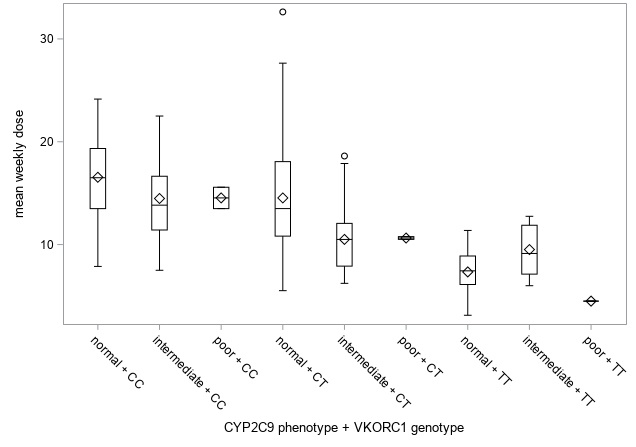
**

The box ranges from the lower quartile to the upper quartile. The line within the box corresponds to the median. The diamond corresponds to the mean. The whiskers range from the smallest to the largest observation within 1.5 times the interquartile range. Circles correspond to any observations outside the inter quartile range.

**Table S5** Average weekly dose per patient grouped by a combination of *CYP2C9* allele carrier status + *VKORC1* genotype

| *CYP2C9* allele carrier status | *VKORC1* genotype | No. of patients | Average weekly dose per patient (mg)  mean ± SD^a^ |
| --- | --- | --- | --- |
| Wildtype | CC | 54 | 16.5±4.0 |
| **2* carrier | CC | 15 | 13.7±4.2 |
| **3* carrier | CC | 7 | 16.1±4.5 |
| Wildtype | CT | 74 | 14.5±5.3 |
| **2* carrier | CT | 18 | 10.8±3.6 |
| **3* carrier | CT | 14 | 10.1±2.3 |
| Wildtype | TT | 17 | 7.3±2.5 |
| **2* carrier | TT | 4 | 10.2±2.7 |
| **3* carrier | TT | 6 | 7.9±2.6 |
| p-value^b^ |  |  | <.0001 |

Based on all available data. ^a^ Four patients without dose information available were excluded. ^b^ p-value based on Jonckheere-Terpstra trend test

**Figure S2** Average weekly dose per patient grouped by a combination of *CYP2C9* allele carrier status + *VKORC1* genotype


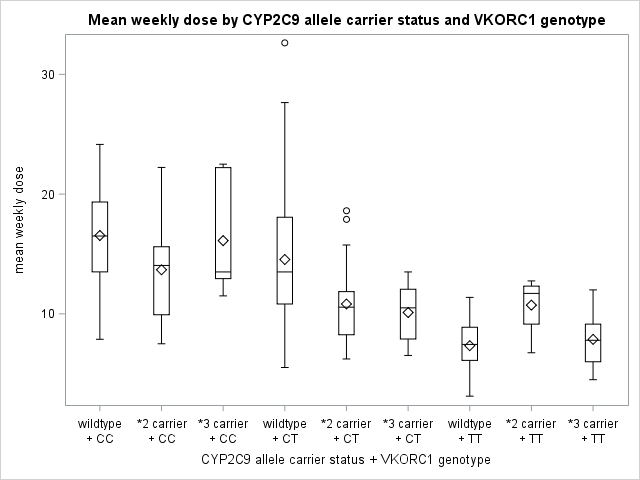


The box ranges from the lower quartile to the upper quartile. The line within the box corresponds to the median. The diamond corresponds to the mean. The whiskers range from the smallest to the largest observation within 1.5 times the interquartile range. Circles correspond to any observations outside the inter quartile range.

**Table S6** Average weekly dose per patient grouped by a combination of *CYP2C9* genotype + *VKORC1* genotype

| *CYP2C9* genotype | *VKORC1* genotype | No. of patients | Weekly dose (mg)  mean ± SD^a^ |
| --- | --- | --- | --- |
| **1/*1* | CC | 54 | 16.5±4.0 |
| **1/*2* | CC | 14 | 13.5±4.3 |
| **1/*3* | CC | 6 | 16.5±4.8 |
| **2/*2* | CC | 1 | 15.6 |
| **2/*3* | CC | 1 | 13.5 |
| **1/*1* | CT | 74 | 14.5±5.2 |
| **1/*2* | CT | 18 | 10.8±3.6 |
| **1/*3* | CT | 12 | 10.0±2.5 |
| **2/*3* | CT | 2 | 10.6±0.2 |
| **1/*1* | TT | 17 | 7.3±2.5 |
| **1/*2* | TT | 4 | 10.7±2.7 |
| **1/*3* | TT | 5 | 8.5±2.3 |
| **2/*3* | TT | 1 | 4.5 |
| p-value^b^ |  |  | <.0001 |

Based on all available data.^a^ Four patients without dose information available were excluded. ^b^ p-value based on Jonckheere-Terpstra trend test

**Figure S3** Average weekly dose per patient grouped by a combination of *CYP2C9* genotype + *VKORC1* genotype


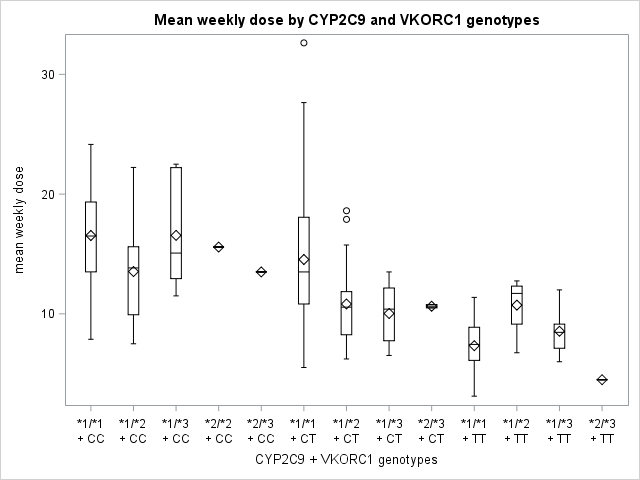


The box ranges from the lower quartile to the upper quartile. The line within the box corresponds to the median. The diamond corresponds to the mean. The whiskers range from the smallest to the largest observation within 1.5 times the interquartile range. Circles correspond to any observations outside the inter quartile range.
